# Does Physical Activity Regulate Prostate Carcinogenesis and Prostate Cancer Outcomes? A Narrative Review

**DOI:** 10.3390/ijerph17041441

**Published:** 2020-02-24

**Authors:** Marco Capece, Massimiliano Creta, Armando Calogero, Roberto La Rocca, Luigi Napolitano, Biagio Barone, Antonello Sica, Ferdinando Fusco, Michele Santangelo, Concetta Dodaro, Caterina Sagnelli, Nicola Carlomagno, Felice Crocetto, Gianluigi Califano, Francesco Mangiapia, Nicola Longo

**Affiliations:** 1 Department of Neurosciences, Reproductive Sciences and Odontostomatology, University of Naples “Federico II”, 80131 Naples, Italyrobertolarocca87@gmail.com (R.L.R.); nluigi89@libero.it (L.N.); biagio193@gmail.com (B.B.); ferdinando-fusco@libero.it (F.F.); felice.crocetto@unina.it (F.C.); gianl.califano2@gmail.com (G.C.); francesco.mangiapia@unina.it (F.M.); nicolalongo20@yahoo.it (N.L.); 2 Department of Advanced Biomedical Sciences, University of Naples Federico II, 80131 Naples, Italy; armando.calogero2@unina.it (A.C.); michele.santangelo@unina.it (M.S.); cododaro@unina.it (C.D.); nicola.anita@tiscali.it (N.C.); 3 Department of Precision Medicine, University of Campania Luigi Vanvitelli, 80131 Naples, Italy; antonellosica@gmail.com; 4 Department of Mental Health and Public Medicine, University of Campania Luigi Vanvitelli, 80131 Naples, Italy; sagnelli.caterina@libero.it

**Keywords:** prostate cancer, elderly, physical activity, cancer prevention, adjuvant treatment

## Abstract

**Background**: Prostate cancer (PCa) represents a common disease in men aged >65 years. The role of physical activity (PA) in patients at risk or diagnosed with PCa represents an evolving issue. We aimed to summarize available evidences about the impact of PA on the pathophysiology and clinical outcomes of PCa. **Methods**: We performed a narrative review. Evidences about the role of PA in elderly patients in terms of PCa biology, epidemiology, oncological and functional outcomes, as well as in terms of impact on the outcomes of androgen deprivation therapy (ADT) were summarized. **Results**: Potential pathophysiological pathways hypothesized to explain the benefits of PA in terms of prostate carcinogenesis include circulating levels of Insulin-like growth factor-1 (IGF-1), oxidative stress, systemic inflammation, sex hormones, and myokines. Clinically, emerging evidences support the hypothesis that PA is associated with decreased PCa risk, improved PCa-related survival, improved functional outcomes, and reduced ADT-related adverse events.

## 1. Introduction

Prostate cancer (PCa) is the most common malignancy among elderly men [1]. The incidence and mortality of PCa worldwide correlate with increasing age, with the average age at the time of diagnosis being 66 years. The probability of developing PCa increases from 0.005% in men younger than 39 years to 2.2% in men aged 40 to 59 years and rises to 13.7% in men aged between 60 and 79 years [1]. Ninety percent of the patients with PCa in the USA are aged 60 years or older [2]. Given the trend toward a progressive aging of the male population and the increase of PCa incidence worldwide, with 1,017,712 new cases (+79.7%) estimated up to 2040, the epidemiological relevance of PCa in elderly patients is growing, and measures of cancer prevention and outcomes improvement are strongly advocated [3]. Potential links have been hypothesized between physical activity (PA) and several PCa issues including incidence, progression, oncological and functional surgical outcomes, and medical therapy-related morbidity [4]. Unfortunately, evidence exists demonstrating that about 11% of older men meet recommended guidelines for PA, and participation decreases as men age [5]. This places men at considerable risk of poor health, including poor cancer outcomes [5]. Exercise interventions can provide benefits in PCa survivors [2]. However, a number of variables exist influencing the impact of physical activity on PCa including exercise typology, intensity, and duration. Herein, we aimed to summarize available evidences of the impact of PA on the pathophysiology and clinical outcomes of PCa, including incidence, survival, functional impairments, and androgen deprivation therapy (ADT)-related adverse events. 

### 1.1. Physical Activity and Prostate Cancer: Pathophysiological Links

**Figure 1:**
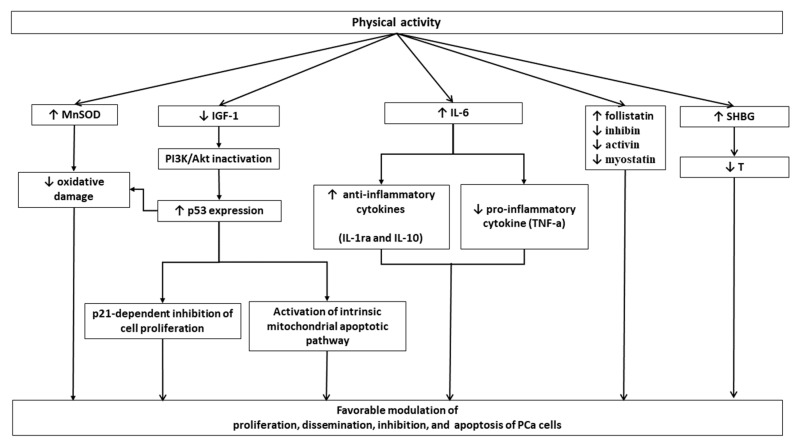
Molecular pathways potentially linking physical activity (PA) and prostate cancer (PCa) carcinogenesis. (IL, interleukin; SHBG: Sex Hormone Binding Globulin; MnSOD: mitochondrial antioxidant manganese superoxide dismutase; T: Testosterone; TNF: Tumor Necrosis Factor.) Evidence exists demonstrating that circulating levels of Insulin-like growth factor-1 (IGF-1), typically associated with chronic hyperinsulinemia, are positively associated with PCa risk, [22]. Indeed, IGF-1 exerts potential pro-neoplastic effects due to the anti-apoptotic activity and the promotion of cell migration [23,24]. Physical activity has the potential to decrease circulating levels of IGF-1 through body fat reduction [23].

Physical activity can directly or indirectly affect all stages of PCa carcinogenesis [6,7,8,9,10,11,12,13,14,15]. Several potential molecular pathways have been proposed to link physical activity and PCa carcinogenesis. These include circulating levels of Insulin-like growth factor-1 (IGF-1), oxidative stress, systemic inflammation, sex hormones, and myokines. These factors are strongly related to each other in complex pathways, as seen in Figure 1 [16,17,18,19,20,21]. 

Moreover, studies have shown that PA might modulate the circulating levels of follistatin, myostatin, activin, and inhibin, which have been reported to be involved in the regulation of proliferation, dissemination, and apoptosis of human PCa cells [24,25]. One hypothetical model considers that follistatin can bind to myostatin or activin, preventing the complex from binding to its receptor and antagonizing their action (inhibition of the apoptosis). It is clear that follistatin neutralizes myostatin activity. However, it is not possible to elucidate all the mechanisms involved in the process, as hundreds of other proteins take part as well and further studies on those are needed [26]. Nevertheless, it has been hypothesized that the induction of growth arrest of PCa cells is regulated by activin, the regulation of PCa cell growth involves myostatin, and the apoptosis of PCa cells depends on activin and its receptor. On the other hand, inhibin and follistatin promote progression of PCa by inhibiting antineoplastic effects of activin and facilitate PCa proliferation via activin receptor. It seems that PA increases plasma levels of follistatin and reduces circulating inhibin, activin, and circulating levels of myostatin [26].

A number of pre-clinical evidences suggest that oxidative stress and antioxidants play a critical role in the pathogenesis of PCa, which is associated with an increase of reactive oxygen species and impaired antioxidant defense mechanisms [27,28]. Regular and moderate PA has the potential to modulate redox signaling and enhancing antioxidant defense, thus decreasing PCa initiation and progression [21,29,30,31,32,33,34,35,36]. Moderate PA can stimulate the production of reactive oxygen species, leading to the activation of ERK1/2 and p 38, which in turn activate NF-ĸB, thus leading to increased expression of antioxidant enzymes such as mitochondrial antioxidant manganese superoxide dismutase (MnSOD) [21,29,30,31,32,33]. Prostate tumorigenesis is associated with systemic low-grade inflammation. Physical activity stimulates interleukin-6 production by muscles that in turn stimulates other systemic anti-inflammatory cytokines such as interleukin-1ra and interleukin-10 and inhibits the synthesis of proinflammatory cytokine such as tumor necrosis factor-a [21]. Some data suggest that telomere shortening is one possible reason why obesity and inactivity may influence PCa or normal tissue. Telomeres are repetitive DNA sequences that protect the ends of chromosomes from degradation and recombination. Telomeres can be shortened, and ultimately become dysfunctional, by incomplete replication during DNA synthesis, alterations of telomere-binding proteins involved in telomere maintenance, or by oxidative stress leading to DNA damage. It seems that PA can modulate protecting enzymes that maintain intact telomeres [34]. It has been hypothesized that the beneficial effects of PA on telomere length are intermediated by a reduction in oxidative stress and chronic inflammation [34].

Physical activity increases levels of sex hormone binding globulin (SHBG), thus negatively modulating free testosterone (T) levels.

However, such studies have never drawn a robust association for many reasons: the long latent phase, the heterogeneity of the studies, and the impact of various external factors that may influence this connection [35,36].

### 1.2. Physical Activity and Prostate Cancer Risk

Studies investigating the relationship between PA and PCa risk provided conflicting and inconclusive results. In their meta-analysis, Liu et al. found a 19% PCa risk reduction for overall PA and a 5% risk reduction for recreational PA. Interestingly, the authors found that PA significantly reduced PCa risk only in the 20–65 age group [37]. Kruk et al. found an overall PCa risk reduction ranging from 5% to 65% associated with recreational PA and from 10% to 56% associated with occupational PA [18]. A large Scandinavian study involving over 1 million patients (73.2% between 60 and 80 years, 18.3% >80 years) reported an overall reduction of PCa rates by 7–12%, for subjects with high perceived physical workload [38]. These results have been subsequently confirmed by Benke et al. who reported a reduction of 17% of PCa risk in patients >60 years with long-term occupational PA [39]. A similar correlation has been reinforced by further studies on body mass index. Patients aged between 60 and 80 with higher body mass index seem to have an increased risk of aggressive PCa at diagnosis [39]. Liu et al. conducted a meta-analysis to investigate the potential relationship between leisure time PA and PCa. Twenty-one cohort studies were included, with a total of 803,872 subjects and 28,707 PCa patients. Authors failed to find association between leisure time activity and risk of PCa, regardless of PCa aggressiveness [40]. More recently, Kazmi et al. assessed whether PA was causally associated with risk of overall or aggressive PCa using Mendelian randomization based on genome-wide association-study summary statistics from the Postate cancer AssoCiation group To Investigate Cancer Associated aLterations (PRACTICAL) and Genetic Associations and Mechanisms of Oncology (GAME-ON)/ Elucidating Loci Involved in Prostate Cancer Susceptibility (ELLIPSE) consortia [41]. Authors found evidence that PA was causally and inversely related to PCa risk [41].

### 1.3. Physical Activity and Prostate Cancer Mortality

There is some evidence that demonstrates improved PCa-related survival in patients who regularly perform PA. Kenfield et al. investigated overall and PCa-specific mortality among 2705 men diagnosed with non-metastatic PCa who lived at least 4 years after their postdiagnosis physical activity assessment. They found that PA was associated with a reduced overall and PCa-specific mortality. In particular, activities such as biking, jogging, tennis, or swimming for ≥3 h a week were associated with a significant improvement of PCa-specific survival [42]. These data have been confirmed by Friedenreich et al., who conducted an observational study on 830 men diagnosed with stage II–IV PCa. They found that post-diagnosis recreational activity (>26 vs. ≤4 metabolic equivalent-hours/week per year) was associated with a significantly lower PCa-specific mortality risk, whereas all-causes-mortality was significantly reduced in patients who regularly performed sustained recreational activity before and after diagnosis (>18–20 vs. <7–8 metabolic equivalent-hours/week per year) [43]. Until 2018 all data on exercise and cancer survival had been retrieved from observational studies; however, Newton et al. designed INTERVAL-GAP4, a multi-centre phase III randomized controlled trial. In this study, 866 patients with documented metastatic PCa were randomly assigned 1:1 to either supervised exercise (high-intensity aerobic and resistance training) or self-directed exercise (provision of guidelines). The trial is still ongoing, but preliminary results support the potential beneficial role of exercise for PCa survival [44].

### 1.4. Physical Activity and PCa-Related Functional Outcomes

Accumulating evidence suggests that PA after PCa diagnosis may improve quality of life (QOL) and psychological and physical outcomes, and reduce cancer-related fatigue, even in patients >65 years [45,46]. Despite this, overall the literature remains equivocal about the possible effects of PA on PCa progression: Recent studies reported a significantly lower prostate-specific antigen (PSA) level and greater physical QOL and social participation in those with higher PA, reducing treatment side effects, fatigue, and incontinence, and furthermore improving the self-perception of masculinity [47,48]. A global increase of 3.85 points was found in the Functional Assessment of Cancer Therapy-Prostate (FACT-P) survey in an exercise intervention group compared to a control group at 12 weeks in patients with a mean age of 69 years in a recent large systematic review [49]. Moreover, PA is feasible and beneficial in patients with bone metastases with no adverse events, skeletal fractures, or increased bone pain reported [50]. It can also improve incontinence in patients undergoing radiotherapy for PCa. Hojan et al. designed a randomized controlled trial in which 27 patients, aged 60 or more, performed supervised, moderate-intensity physical exercise (active group) and another 27 carried out normal daily PA (control group). After radiotherapy, there was a significant improvement in functional outcomes in the active group, as well as fatigue perception and overall quality of life [51,52,53].

Phillips et al. prospectively examined the associations between post-diagnosis activity and QOL domains in men diagnosed with non-metastatic PCa in the Health Professionals Follow-up Study [52]. Elderly patients diagnosed with non-metastatic PCa and receiving whichever treatment may benefit from PA in terms of urinary continence and sexual function. Those who walk at a brisk pace have a statistically significant reduction of urinary incontinence (85.8 vs. 80.1, *p* = 0.01) and a better sexual function (37.3 vs. 29.1, *p* = 0.001) compared to men who walk at an easy pace, regardless of walking duration. Furthermore, patients who walk ≥90 min at a normal to very brisk pace report better vitality scores (88.5 vs. 90.3, *p* = 0.001) than those who walk less. By contrast, results for weightlifting are conflicting, as it seems that weightlifting is associated with increased urinary incontinence [52,54].

### 1.5. Physical Activity and Androgen Deprivation Therapy—Related Toxicity

Androgen deprivation therapy is associated with several adverse events including reduced bone mineral density, loss of muscle mass, and increased body fat mass [55,56].

Evidence exists demonstrating that regular PA may counteract the effects of ADT, improving mental and physical health and enhancing quality of life in men with PCa. Bourke et al. demonstrated that lifestyle intervention, including physical exercise, improved ADT-related fatigue in a randomized controlled study. Twenty-five patients were advised to follow a 12-week lifestyle program comprising aerobic and resistance exercise plus dietary advice and were compared to 25 patients who received standard care. The intervention group massively improved exercise behavior fatigue and muscle strength [57,58]. Taaffe et al. found that the higher the fatigue experienced by the patients, the more pronounced the benefit of a lifestyle intervention [59].

Although PA has the potential to improve body composition secondary to the severe hypogonadism induced by ADT, Cormie et al., in a randomized controlled trial, showed that fat mass increase during the first three months of ADT can be prevented by a twice-weekly moderate-to-high exercise session [60].

Less strong is the relationship between physical exercises and bone density response. Mennen-Winchell et al. investigated the effects of self-reported daily PA on bone density of the hip and spine measured with dual-energy X-ray absorptiometry in patients receiving ADT. The authors concluded that increased endurance exercise may help in prevention of low bone density of the hip, but not of the spine [61]. Moreover, not all bone sites have been investigated in the literature; thus, further studies are needed to elucidate the impact of PA on bone health.

Benefits of physical exercise are also evident in libido and potency. Indeed, sexual function decline during ADT seems to be attenuated in patients that regularly work out. Hamilton et al. found that exercise reduced body feminization by increasing muscle mass and blocking weight gain. Moreover, the ability to perform exercises improved masculine self-esteem and allowed the patients to shift their focus away from sexual dysfunction and to embody idealized masculinity through physical performance. Thus, PA may help to manage the negative impact of ADT on male sexuality [62,63].

Interestingly, a recent meta-analysis performed by Chen et al. evaluating the effects of supervised exercise on muscle mass and strength in cancer patients undergoing ADT failed to find statistically significant changes in terms of total lean mass in subjects undergoing supervised exercise compared to subjects undergoing routine care. Thus, the benefits of physical exercise do not necessarily alter lean mass in patients receiving ADT. A possible explanation for such discrepancy may be related to testosterone suppression. In fact, testosterone is the most important androgen that controls muscle protein synthesis; thus, in such patients the muscle growth induced by intensive training is abolished too [64].

## 2. Discussion

Older men diagnosed with PCa represent a vulnerable population due to comorbidities, cancer itself, and cancer-related treatments. In recent years, many data have emerged demonstrating the association between PA and PCa risk, and basic research has been conducted to clarify the pathophysiological link. Although detailed biological mechanisms are not completely understood, several potential biological pathways have been hypothesized to mediate the beneficial effects of PA on PCa biology.

Some studies have hypothesized that PA may also prevent PCa; however, the quality of those is rather low and there is a conflicting evidence regarding this point. Therefore, a definitive postulate cannot be stated. On the other hand, it is quite clear that PA in older men who have undergone curative treatment for PCa improve either quality of life, and functional outcomes (such as erectile dysfunction and urinary incontinence), and oncological outcomes (increasing overall survival and cancer-specific survival). The present review, despite the huge amount of studies found on the subject, has few outstanding limitations. First, despite the growing number of elderly patients suffering from this type of cancer, the overall quality of the studies found in the literature is scarce. Most of these studies are observational, retrospective, with a poor methodology and with a very low number of patients; thus, the strength of our findings is limited. Moreover, another limitation of the present literature is related to the different PA protocols and the various ways of measuring the effects of such therapies (i.e., questionnaires, self-evaluation, muscle mass, etc.). To date a clear and robust recommendation cannot be provided. Further pre-clinical studies are needed to elucidate molecular and cellular key signaling pathways linking PA and PCa. From a clinical point of view, more robust and methodologically correct randomized controlled trials are needed to confirm existing data. Based on current evidence, however, older patients suffering from PCa should be counselled about the potential benefits of PA.

## 3. Conclusions

Physical activity has the potential to favorably influence the pathophysiology and outcomes of PCa in elderly subjects. Further studies are needed to confirm these evidences.
